# Unraveling the metabolic network of organic acids in solid‐state fermentation of Chinese cereal vinegar

**DOI:** 10.1002/fsn3.2409

**Published:** 2021-06-18

**Authors:** Yanfang Wu, Menglei Xia, Xiaofeng Zhang, Xiaowei Li, Rongzhan Zhang, Yufeng Yan, Fanfan Lang, Yu Zheng, Min Wang

**Affiliations:** ^1^ State Key Laboratory of Food Nutrition and Safety Key Laboratory of Industrial Fermentation Microbiology Ministry of Education College of Biotechnology Tianjin University of Science & Technology Tianjin China; ^2^ Shanxi Province Key Laboratory of Vinegar Fermentation Science and Engineering Shanxi Zilin Vinegar Industry Co., Ltd. Taiyuan China; ^3^ Tianjin Tianli Duliu Mature Vinegar Co., Ltd. Tianjin China

**Keywords:** cereal vinegar, metabolic network, metagenomics, organic acid, solid‐state fermentation

## Abstract

Shanxi aged vinegar (SAV) is fermented by multispecies microorganism with solid‐state fermentation (SSF) technology, which contains a variety of organic acids. However, the metabolic network of them in SSF is still unclear. In this study, metagenomics technology was used to reveal the microbial community and functional genes in SAV fermentation. The metabolic network of key organic acids with taste active value higher than 1 was reconstructed for the first time, including acetate, lactate, malate, citrate, succinate, and tartrate. The results show pyruvate is the core compound in the metabolic network of organic acids. Metabolic pathway of acetate plays a pivotal role in this network, and acetate has regulatory function on metabolism of other organic acids. *Acetobacter* and *Lactobacillus* are the predominant genera for organic acid metabolism in SSF of SAV. This is also the first report on metabolic network of organic acids in cereal vinegar, adding new knowledge on the flavor substance metabolism during multispecies fermentation of traditional fermented food.

## Graphical abstract

Metagenomics technology was used to reveal the microbial community and functional genes in Chinese cereal vinegar. The metabolic network of key organic acids with taste active value higher than 1 was reconstructed for the first time. Acetobacter and Lactobacillus are the predominant genera for organic acid metabolism in solid‐state fermentation of vinegar.

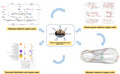

## 1 INTRODUCTION

Vinegar, as a traditional fermented food, is an indispensable condiment in the world. Shanxi aged vinegar (SAV) is known as one of the most famous vinegar in China, which is fermented with solid‐state fermentation (SSF) technology with sorghum as the main raw material (Tang et al., 2019). Organic acids are the key flavor compounds, contributing unique characteristics of SAV (Chen et al., 2016). The composition of organic acids is one of the main differences of diverse kinds of vinegar. It was reported that a total of 32 kinds of organic acids were determined in cereal vinegar with SSF, including acetate, lactate, oxalate, malate, citrate, succinate, and tartrate (Li et al., 2016). In contrast, there are fewer kinds of organic acids in vinegar with liquid fermentation (Budak & Guzel‐Seydim, 2010; Štornik et al., 2016; Trček et al., 2016). The formation of organic acids of vinegar mainly depends on the metabolism of microorganisms (Li et al., 2015). Abundant microorganisms involved in SSF of cereal vinegars are an important reason for the diversity of organic acids (Nie et al., 2017). Recently, studies have focused on the correlation between organic acids in vinegar and microbial community, and inferred significantly related microorganisms (Nie et al., 2017). The network of flavor substances at pathway level in vinegar was predicted (Wu et al., 2017), which gives a preliminary understanding of the organic acid formation. However, the metabolic relationship between enzyme genes and related compounds, and the distribution of microbiota for organic acid formation are still unknown. Therefore, it is necessary to construct a detailed metabolic network of the organic acids in vinegar fermentation.

KEGG (Kyoto Encyclopedia of Genes and Genomes) pathway data are encoded in KGML (KEGG Markup Language) format, providing information of enzymes, compounds, and reactions (Kanehisa et al., 2007). Based on this, the organism‐specific substance metabolic network could be reconstructed in accordance with requirements (Zhou, 2013). In this work, on the basis of annotation of functional genes and enzymes revealed by metagenomic technology, the metabolic network of organic acids in SAV was reconstructed at the enzyme level. In addition, the distribution of related microorganisms was also explored. This study sheds new light on visual understanding of the flavor substance metabolism during multispecies fermentation of traditional fermented food.

## 2 MATERIALS AND METHODS

### 2.1 Sampling

Acetic acid fermentation (AAF) samples (*Cupei*) and vinegar samples of SAV were collected from Taiyuan, China. Samples on day 1, day 3, day 5, day 7, and day 9 represent the early stage, middle‐early stage, middle stage, middle‐late stage, and late stage of fermentation, respectively. Samples were collected at a depth of 30 cm from the surface of *Cupei*. Samples for each day were collected from 3 parallel vats. The metagenomic sequencing of each sample was carried out respectively, and subsequent library was constructed from all sequencing information of five *Cupei* samples. Vinegar samples were collected after the leaching step to obtain the concentrations of organic acids.

### 2.2 Determination of organic acids in vinegar

Samples of 5.0 ml vinegar were added to 45.0 ml dH_2_O and subjected to centrifugation at 2,102.7 *g* for 5 min for determination. Samples after the pretreatment were filtered with 0.45 μm microporous membrane. A high‐performance liquid chromatography (HPLC) (Agilent) system equipped with an Aminex HPX‐87H 300 × 7.8 (mm) column (Bio‐Rad) was used for identification and analysis of organic acids. Mobile phase: 5 mmol/L H_2_SO_4_; flow rate: 0.6 ml/min; injection volume: 20 μl; UV detector wave length: 215 nm; column temperature: 30°C. Organic acid standards (acetate, lactate, citrate, malate, oxalate, succinate, and tartrate) were from Sigma‐Aldrich. The measurement was taken in triplicate. The taste active value (TAV) was calculated as the ratio between the concentrations of organic acids measured above and its threshold value in vinegar (Duan et al., 2020).

### 2.3 DNA extraction and metagenomic sequencing

2 g *Cupei* samples of each day were mixed and placed in a sterile mortar and rapidly ground to powder by adding liquid nitrogen. Genomic DNA was extracted from about 500 mg of the sample using the previous method (Nie et al., 2015). The preparation of the next‐generation sequencing library was in accordance with the manufacturer's protocol of NEBNext Ultra DNA Library Prep Kit (Illumina). After multiplexing the libraries with different indexes, 2 × 150 paired‐end sequencing was performed according to Illumina Hiseq (Illumina, San Diego, CA, USA) instructions. The sequence information was read by Hiseq Control Software +OLB +GAPipeline‐1.6 (Illumina) on the Hiseq instrument.

Cutadapt (v1.9.1) (https://pypi.org/project/cutadapt/1.9.1/) was used to remove low‐quality reads, N‐rich reads, adapter‐polluted reads and host contamination reads sequences from the pass filter data. Based on the clean data, assembly analysis was carried out using SOAPdenovo (v2) (Li et al., 2010) with different k‐mer. Scaffold with the largest N50 was selected as the final assembly result for subsequent analysis. CD‐HIT (v4.5.6) (Fu et al., 2012) was used to cluster scaftigs derived from assembly with a default identity of 0.95.

The *Cupei* metagenomic sequencing data have been uploaded to NCBI Short Read Archive, and the accession number is PRJNA689964.

### 2.4 Functional and taxonomy annotation of unigenes

Based on the predicted protein sequences of coding genes, BLAST software (version 2.2.31+) was used to compare with the protein sequences by running local blast against the nr database. The cutoffs used were 60% coverage on the profile with an *e*‐value better than 10^−5^. MEGAN (MEtaGenome ANalyzer) (Huson et al., 2011) was used to analyze the taxonomic profiles using the lowest common ancestor (LCA) algorithm.

### 2.5 Reconstruction of organic acid metabolism network

Metabolic network was reconstructed from the enzyme‐compound relationship information. The KGML data of pathway ko00010, ko00620, ko00020, ko00630 ko00250, and ko00720 related to organic acid metabolism were downloaded from https://www.genome.jp/kegg/pathway.html. Afterward, KGML data only containing annotated enzymes revealed from metagenomic sequencing and corresponding compounds and reactions were imported into Cytoscape 3.7.2 software and presented as a network. All the edges were directional in the network and the arrow represented the reaction direction. Duplicate edges including “self‐loops” were removed. Topological analyses were performed by employing “Network Analyzer” plugin of Cytoscape.

## 3 RESULTS AND DISCUSSION

### 3.1 Organic acids in SAV

In the present study, 7 kinds of common organic acids, including oxalate, citrate, tartrate, malate, succinate, lactate, and acetate were identified in SAV (Table 1), which was consistent with the previous study (Kong et al., 2017; Li et al., 2016). Acetate is the dominant organic acid in vinegars and occupied 55.28% of total organic acid content, which has pure acetate stimulation, short aftertaste, and poor seasoning effect (Kong et al., 2017). Lactate is the 2nd largest amount of organic acid in the SAV and occupied 39.09% of the total organic acid content. As the nonvolatile organic acid with the highest content in vinegar, lactate plays a role together with other nonvolatile organic acids to neutralize the stimulation of acetate and provided vinegar with a soft flavor (Yu et al., 2018).

**TABLE 1:** The contents and TAVs of organic acids in SAV

Organic acids	Contents (g/100 ml)	Taste threshold (g/100 ml)^a^	TAV
Oxalate	0.069 ± 0.031	0.050	1.38
Citrate	0.054 ± 0.002	0.045	1.20
Tartrate	0.110 ± 0.028	0.002	55.00
Malate	0.145 ± 0.044	0.050	2.90
Succinate	0.040 ± 0.027	0.011	3.64
Lactate	2.900 ± 0.811	0.126	23.02
Acetate	4.101 ± 1.160	0.011	372.82

^a^ Taste threshold value (g/100 ml) (Kato et al., 1989; Kong et al., 2017).

Compounds with TAV ≥1 were considered to be responsible for taste, and the greater the TAV is, the more contribution to the taste profile (Duan et al., 2020). As listed in Table 1, organic acid that plays a major role in the overall taste of SAV is acetate due to its highest TAV. Specifically, tartrate has an extremely low threshold, so its TAV is as high as 55. In addition, the TAVs of the other 5 organic acids are all higher than 1, indicating that each of these organic acids has significant effects on the taste of vinegar. These various organic acids with different proportions and tastes provide a unique taste for vinegar (Jiang et al., 2019).

### 3.2 Overview of metagenomic data and functional annotations

Totally 127.6 Gbp sequence files were resulted from Illumina Hiseq sequencing of metagenome, and 83,968 contigs were generated by MetaVelvet, with a maximum contig length of 97,470 bp and a minimum contig length of 200 bp. The N50 was 935 bp. The general assembly features of metagenomic sequence are described in Table S1.

**FIGURE 1:**
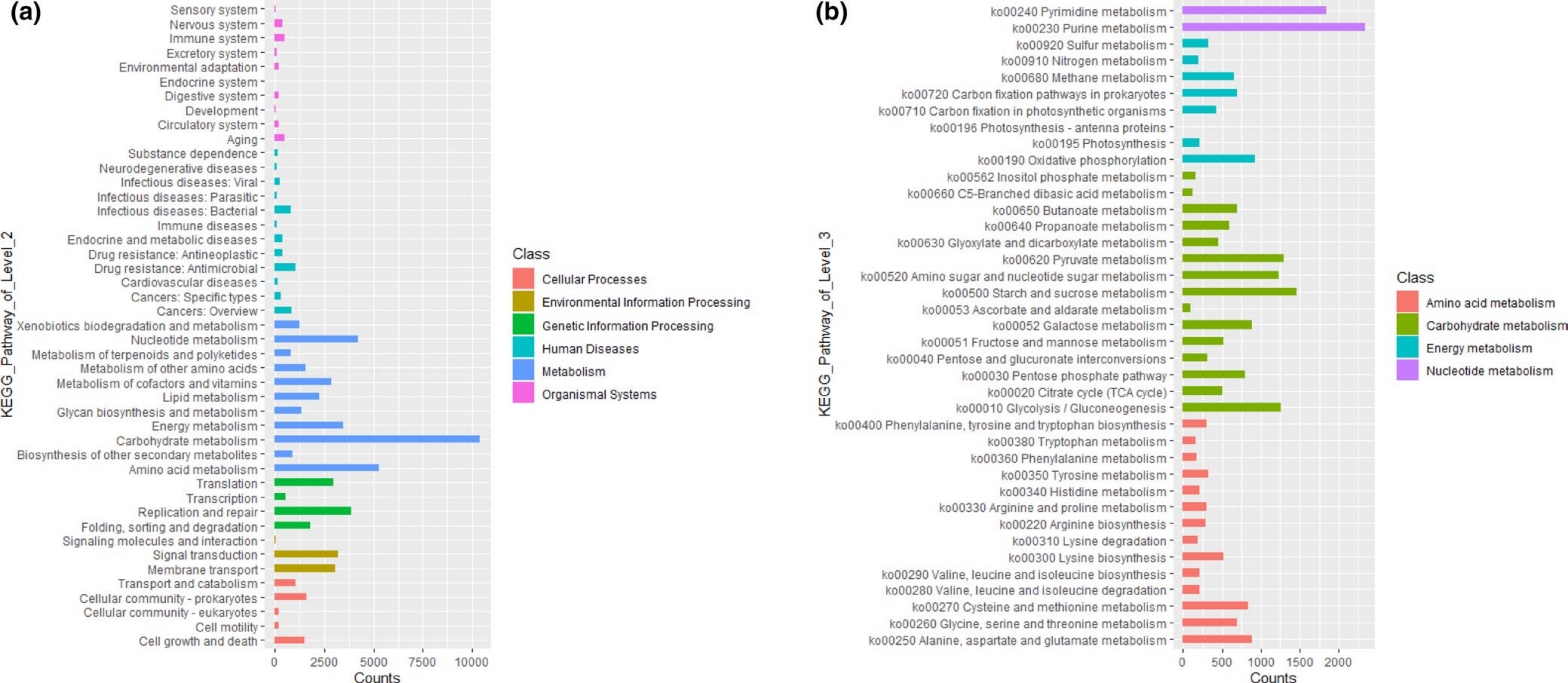
Functional gene categories. Level 1, Level 2 (a), and Level 3 (b) based on KEGG

A total of 60,648 unigenes were annotated with KEGG pathways, among which 34,272 (56.51%) belong to Metabolism group, presenting the highest proportion. In addition, 4,428 (7.30%) belong to Cellular Processes, 6,227 (10.35%) belong to Environmental Information Processing, 9,205 (15.01%) belong to Genetic Information Processing, 4,473 (7.38%) belong to Human Diseases group, and 2,093 (3.45%) belong to Organismal Systems (Figure 1a). At the Level 2 classification level, carbohydrate metabolism has a proportion of 17.18%, presenting the highest enrichment. Furthermore, carbohydrate metabolism accounts for 30.40% in Metabolism group, which is followed by amino acid metabolism, nucleotide metabolism, and energy metabolism, accounting for 15.44%, 12.21%, and 10.12%, respectively.

Further analysis of the 4 most enriched pathways shows that in carbohydrate metabolism, functional genes are mainly enriched in ko00500 Starch and sucrose metabolism, ko00620 Pyruvate metabolism, ko00010 Glycolysis/Gluconeogenesis, and ko00520 Amino sugar and nucleotide sugar metabolism pathways (Figure 1b). It provides evidence for the efficient decomposition and utilization of polysaccharides by microorganisms and the formation of diverse flavor substances in the process of vinegar fermentation (Wu et al., 2017). Corresponding to the previous recognition on the various types and contents of amino acids in vinegar (Nie et al., 2017; Wang et al., 2015), functional genes are mainly enriched in amino acid metabolism in ko00250 Alanine, aspartate, and glutamate metabolism, ko00270 Cysteine and methionine metabolism, ko00260 Glycine, serine and threonine metabolism, and ko00300 Lysine biosynthesis. Additionally, ko00230 Purine metabolism and ko00240 Pyrimidine metabolism, probably more related to cell growth, are enriched in nucleotide metabolism, indicating a rapid accumulation of microorganisms during AAF process (Nie et al., 2017). Specifically, oxidative phosphorylation provides the driving force for the majority of respiratory ATP generate (Hoelzle et al., 2014). In present study, ko00190 Oxidative phosphorylation is the pathway involving the highest number of functional genes in energy metabolism, indicating that microorganisms metabolized rapidly and released a large amount of energy to drive the energy‐demanding reaction due to the stress condition during AAF of SAV (Zheng et al., 2018).

### 3.3 Taxonomic classification of predicted genes

**FIGURE 2:**
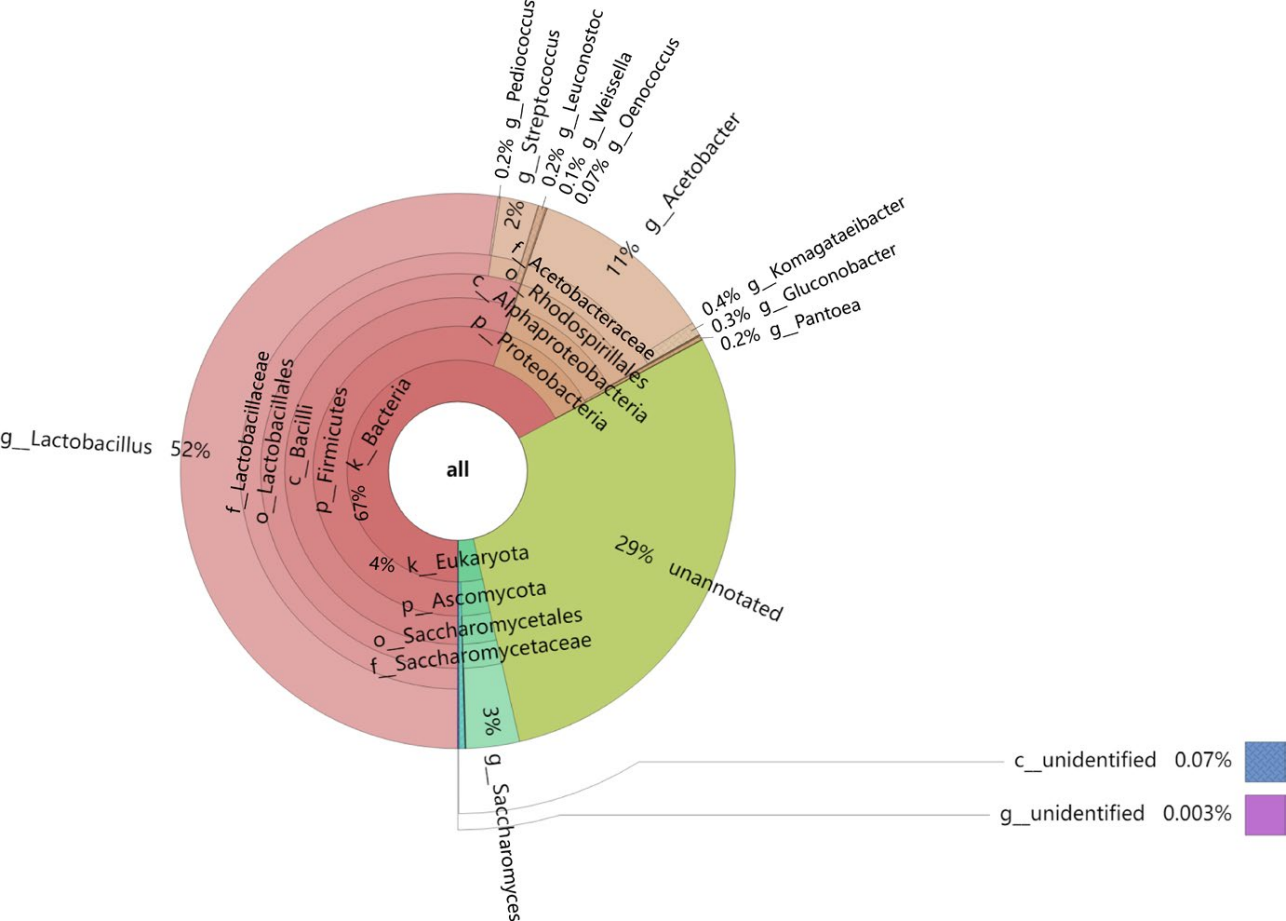
Krona chart of taxonomic affiliation of microbiota and their relative abundance. Inner circles represent higher taxonomic ranks, and more detailed taxonomic ranks (up to species level) are presented in outer circles

The composition of microorganisms in the AAF of SAV has been identified by culture and nonculture methods (Nie et al., 2015; Nie et al., 2017; Wu et al., 2012; Zheng et al., 2018). In this study, microbial community in fermentation process was characterized through metagenomic sequencing technology. The unigenes obtained by Illumina Hiseq sequencing were compared with the 16S rDNA database, and the OTUs were classified into 186 genera. Taxonomic Krona classification (Figure 2) shows the community distribution at each classification level. On the whole, the microorganisms in *Cupei* are mainly bacteria, with an abundance of 67%, while Eukaryota accounts for only 4%, and 29% of all was not annotated. Firmicutes is the main phylum of bacteria, followed by Proteobacteria, which is consistent with the results of high‐throughput sequencing (Zhu et al., 2018). There is no doubt that *Lactobacillus* is the most abundant in Firmicutes, accounting for 52% of the total microorganisms, and it is one of the predominant genera in *Cupei*. In addition, *Streptococcus* (2%), *Pediococcus* (0.2%), *Leuconostoc* (0.2%), *Weissella* (0.1%), and *Oenococcus* (0.07%) were all detected among Firmicutes.

As the key microorganism of AAF, *Acetobacter* is the most abundant genus in the phylum of Proteobacteria, accounting for 11% of the total microbial abundance, and it is the second most abundant genus in SAV fermentation (Figure 2). Among Proteobacteria, *Komagataeibacter* (0.4%), *Gluconobacter* (0.3%), and *Pantoea* (0.2%) also show relatively high abundance, which is similar to the previous study (Nie et al., 2017).

Regarding to Eukaryota, Ascomycota is the main phylum in SAV fermentation, and *Saccharomyces* accounts for 3% of the total microbial abundance, resulting in a dominant genus of Eukaryota. Overall, our results conclude that there exist abundant microorganisms in *Cupei* of SAV, which contribute to SSF process together, and the main genera are *Lactobacillus*, *Acetobacter,* and *Streptococcus*.

### 3.4 Reconstruction of metabolic network of organic acids in SAV

According to KEGG database and the metagenomic annotations, the degradation of substrates and metabolic pathways of major organic acids in microbial community during AAF of SAV were predicted (Figure S1). The raw materials for the AAF of SAV include bran, rice husk, and ethanol from *Jiulao* (mash gained from alcohol fermentation). Glucose and other monosaccharides, oligosaccharides, and alcohols obtained from the degradation of starch and cellulose in *Cupei* by microorganisms can be used as carbon sources (Tang et al., 2019). Specifically, ethanol is not only the flavor compound (Wang et al., 2015), but also the key substrate of acetate. In addition, the nitrogen source in vinegar fermentation is derived from amino acids and inorganic sources such as nitrate and nitrite (Wu et al., 2017). Reportedly, most organic acids are produced in fermentation, while minors derive from raw materials (Kong et al., 2017). It should be noticed that the enzyme genes related to oxalate metabolism were not found in the metagenomics of SAV, indicating that the metabolism of oxalate was not carried out in microbial community. It is generally believed that oxalate is ubiquitous in plants (Kayashima & Katayama, 2002). Among the raw materials of AAF, bran was proven to be rich in oxalate (Jahnen et al., 1992). Therefore, oxalate in SAV is mainly derived from raw materials. Beyond that, acetate, lactate, malate, citrate, tartrate, and succinate could be produced by microbial metabolism in SSF (Figure S1). The pathways of acetate metabolism are composed of ko00010 (glycolysis/gluconeogenesis) and ko00620 (pyruvate metabolism). The pathway of lactate metabolism is ko00620 (pyruvate metabolism). The pathways of malate metabolism are composed of ko00020 (TCA cycle), ko00620 (pyruvate metabolism), and ko00630 (glyoxylate and dicarboxylate metabolism). The pathways of citrate metabolism are composed of ko00020 (TCA cycle) and ko00630 (glyoxylate and dicarboxylate metabolism). The pathway of tartrate metabolism is ko00630 (glyoxylate and dicarboxylate metabolism). The pathways of succinate metabolism are composed of ko00020 (TCA cycle), ko00250 (alanine, aspartate, and glutamate metabolism), ko00630 (glyoxylate and dicarboxylate metabolism), and ko00720 (carbon fixation pathways in prokaryotes). Annotated enzymes of the above pathways are shown in Figures S2–S7.

**FIGURE 3:**
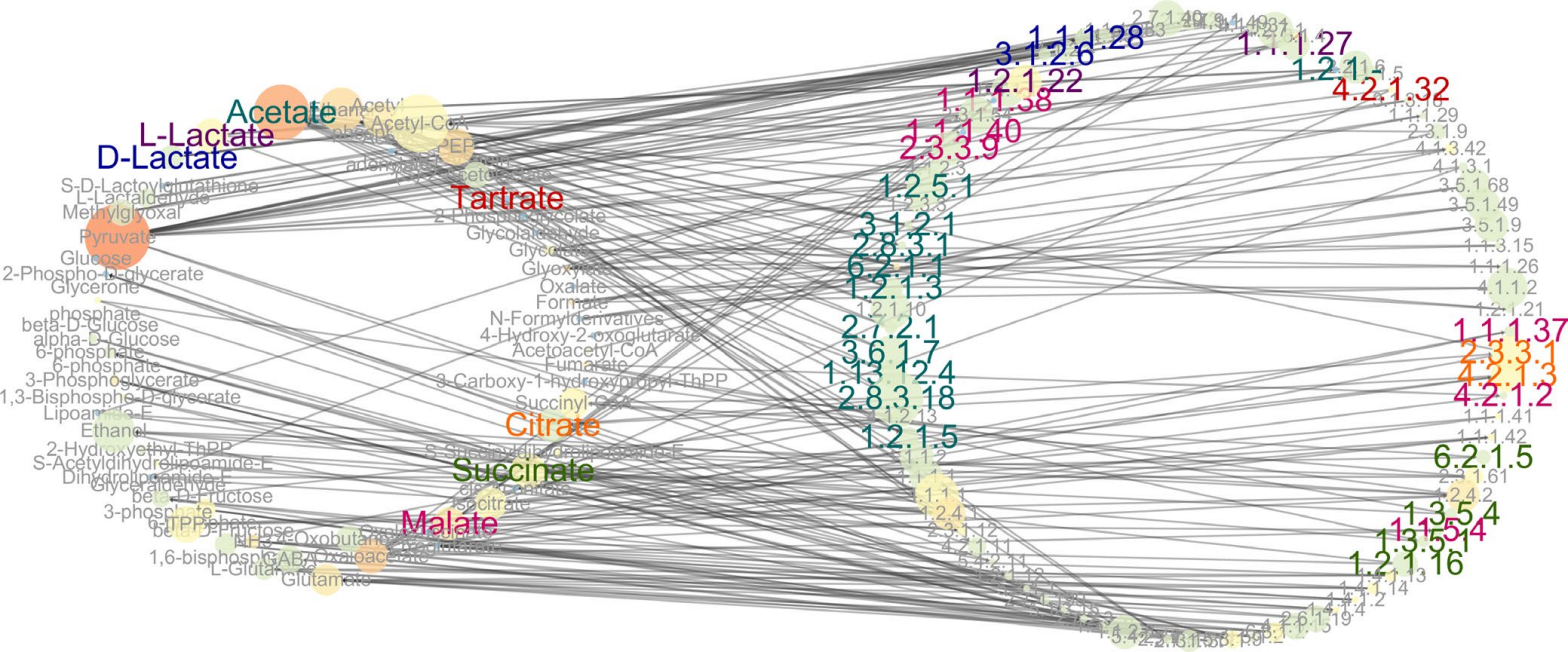
Metabolic network of main organic acids in the microbial community. Circle size represents the betweenness centrality and color brightness of circle represents the in‐degree. Each organic acid and the enzymes related to are marked in font with same color. EC: 2.8.3.18 (succinyl‐CoA: acetate CoA‐transferase) is not only an enzyme of acetate metabolism, but also an enzyme of succinate

In order to further analyze the organic acid metabolism of SAV, the main organic acid metabolic network (OAMN), including acetate, lactate, malate, citrate, tartrate, and succinate in the fermentation process was reconstructed (Figure 3). This OAMN presents the metabolic pathways and enzymes and is composed by 146 nodes linked by 206 directed edges. 57 compounds and 89 enzymes are involved in the metabolism of 7 organic acids, and there are 206 relational chains among them. Those results indicate that the OAMN in SSF of SAV is quite complex due to the diversity of microorganisms.

**FIGURE 4:**
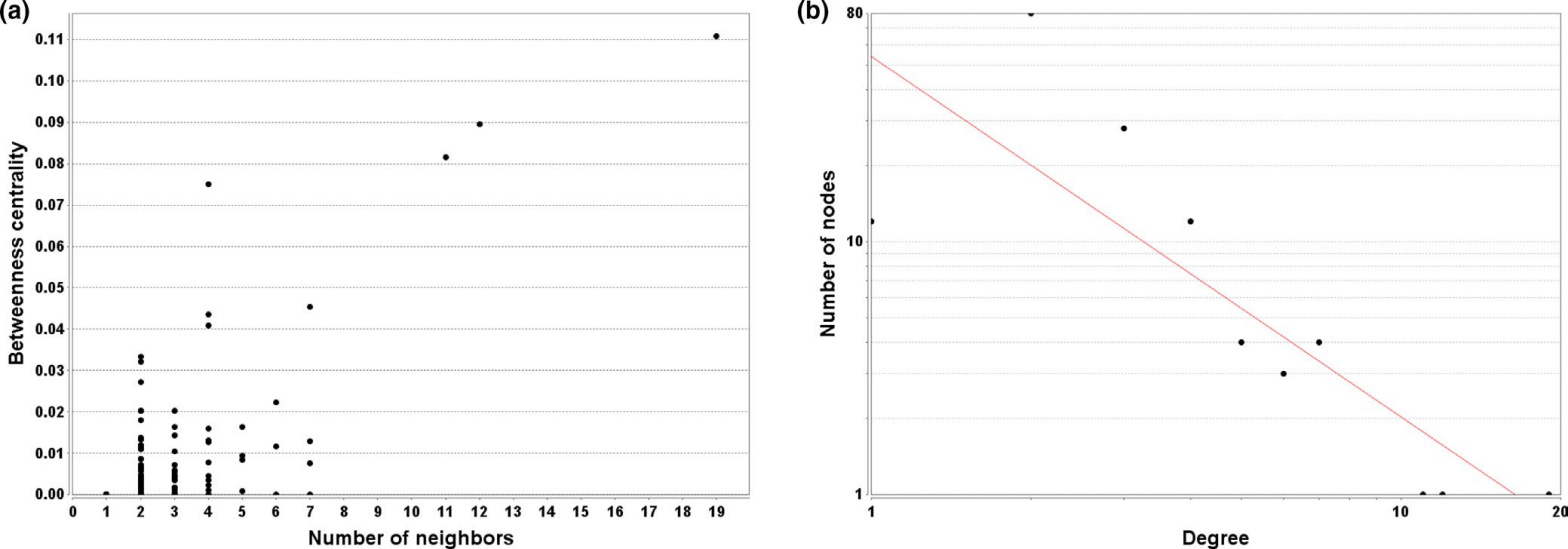
Topological attributes of the network. (a) Betweenness centrality of the network. (b) Node degree distribution of the network with power fitted

Two significantly topological parameters named betweenness centrality and degree were selected as the guidelines for screening the most influential nodes (compounds and enzymes). Betweenness centrality, an index reflecting the node's centrality in network, was measured by the number of shortest paths through a node (Wu et al., 2019), and a high value represents large control over the network. Figure 4a explains that 4 nodes with betweenness centrality are greater than 0.05, including compound nodes of pyruvate, acetyl‐CoA, acetate, and enzyme node of succinyl‐CoA: acetate CoA‐transferase (EC: 2.8.3.18) (Table S2). Moreover, highest betweenness centrality in OAMN is pyruvate with the value of approximately 0.11, and in that case, the number of neighbors is 19. This result provides the evidence that pyruvate is the core compound in OAMN. Similar results were also found in the flavor metabolic network of the other cereal vinegar (Wu et al., 2017). Therefore, pyruvate plays an important role in the microbiota metabolism in SSF of SAV, especially organic acid metabolism. The degree of a given node is defined as the number of edges that directly connect to the node. The power law of node degree distribution P(*k*) was used to evaluate the robustness of OAMN (Barabasi & Oltvai, 2004). It has been reported that the exponent form of the power law in any scale‐free biological network should be less than 2 (Karthikeyan et al., 2016). In this study, the P(*k*) of OAMN followed an exponential law (*y* = *ax^b^*), whose exponent *b* was −1.423 and *R* ^2^ was .703 (Figure 4b), indicating the network possesses a significantly scale‐free property (Barabasi & Oltvai, 2004). In other words, OAMN is not a random network but entirely a definite one.

In OAMN, in‐degree of each organic acid represents the number of enzymes that directly relate to its metabolism (as listed in Table S3). As shown in the network (Figure 3), enzymes involved in the metabolism of acetate are the most abundant, with an amount of 11. Among the various pathways of acetate metabolism in SAV, acetyl‐CoA pathway involves the most abundant enzymes, including propionate CoA‐transferase (EC: 2.8.3.1), acetyl‐CoA synthetase (EC: 6.2.1.1), acetyl‐CoA hydrolase (EC: 3.1.2.1), and succinyl‐CoA: acetate CoA‐transferase (EC: 2.8.3.18) (Figure 3, and Figure S8). Specifically, succinyl‐CoA: acetate CoA‐transferase (EC: 2.8.3.18) is the node of enzyme with the highest betweenness centrality of all enzymes (Table S2), indicating that succinyl‐CoA: acetate CoA‐transferase is the core enzyme in OAMN, catalyzing the freely reversible transfer of CoA between succinate and acetate (Figure 3). Moreover, succinyl‐CoA: acetate CoA‐transferase is present in acetic acid bacteria (AAB) and identified as an enzyme responsible for the assimilation of acetate involve in the TCA cycle (Fukaya et al., 1993). These results provide one of the most crucial evidences for AAB survival in SAV fermentation. Ethanol is the main substance for the formation of acetate during vinegar fermentation, which is catalyzed by ethanol dehydrogenase to produce acetaldehyde and then acetate by aldehyde dehydrogenases (Wu et al., 2017). As a precursor of acetate, acetaldehyde is also catalyzed by a variety of enzymes of aldehyde dehydrogenases, including EC: 1.2.1.3, EC: 1.2.1.5, and EC: 1.2.1.‐ (Figure 3, and Figure S8). We noticed that acetyl phosphate could also form acetate under the catalysis of acetyl phosphatase (EC: 3.6.1.7) and acetate kinase (EC: 2.7.2.1) (Figure S8). Furthermore, pyruvate is another substrate for acetate formation, in which pyruvate dehydrogenase (quinone) (EC: 1.2.5.1) is involved. In addition to these substances, acetate can also be formed through L‐lactate catalyzed by lactate 2‐monooxygenase (EC: 1.13.12.4) (Figure 3, and Figure S8), which might be one of the reasons for the decrease in lactate during SAV fermentation (Zhang et al., 2020). Therefore, the acetate metabolism has an effect of regulatory on other organic acid. Moreover, acetate is identified as hub organic acid due to its highest degree of connection (Table S3). Those results indicate that metabolic pathway of acetate plays a pivotal role in this network.

Lactate in vinegar includes L‐lactate and D‐lactate with a comparable content (Chai et al., 2020), which are formed from pyruvate, lactaldehyde, and S‐D‐lactoylglutathione in SSF of SAV through lactate dehydrogenase (EC: 1.1.1.27 and EC: 1.1.1.28), lactaldehyde dehydrogenase (EC: 1.2.1.22), and hydroxyacylglutathione hydrolase (EC: 3.1.2.6), respectively (Figure 3, and Figure S8). It should be noted that lactate can be also converted into pyruvate that is the precursor of other organic acids and flavor substances (Figure 3) (Wu et al., 2017). As an intermediate substance, acetyl‐CoA participates the TCA cycle and is the central compound of network other than pyruvate (Figure 3) (Table S2). It is also involved in the metabolism of malate and citrate through malate synthase (EC: 2.3.3.9) and citrate synthase (EC: 2.3.3.1), respectively. In addition to acetyl‐CoA, pyruvate, fumarate, oxaloacetate, and glyoxylate are also the precursors of malate. There are 6 enzymes involved in total, including malate synthase (EC: 2.3.3.9), malate dehydrogenase (EC: 1.1.1.37, EC: 1.1.1.38, EC: 1.1.1.40 and EC: 1.1.5.4), and fumarate hydratase (class I) (EC: 4.2.1.2) (Figure 3, and Figure S8). In contrast, the precursor of citrate, except acetyl‐CoA, is only isocitrate and oxaloacetate, and 2 enzymes of aconitate hydratase (EC: 4.2.1.3) and citrate synthase (EC: 2.3.3.1) are involved (Figure 3, and Figure S8). Succinyl‐CoA, fumarate, and succinic semialdehyde are the precursors of succinate. The number of enzymes related to succinate ranked third, behind that of acetate and malate. These enzymes are succinyl‐CoA: acetate CoA‐transferase (EC: 2.8.3.18), succinyl‐CoA synthetase alpha subunit (EC: 6.2.1.5), fumarate reductase flavoprotein subunit (EC: 1.3.5.4), succinate dehydrogenase (ubiquinone) flavoprotein subunit (EC: 1.3.5.1), and succinate‐semialdehyde dehydrogenase (EC: 1.2.1.16) (Figure 3, and Figure S8). Reportedly, tartrate can be formed by 3 enzymes of tartrate epimerase (EC: 5.1.2.5), tartrate dehydrogenase (EC: 1.1.1.93), and L (+)‐tartrate dehydratase alpha subunit (EC: 4.2.1.32) (Hurlbert & Jakoby, 1965; Kohn et al., 1968; Ranjan et al., 1961). However, tartrate epimerase (EC: 5.1.2.5) and tartrate dehydrogenase (EC: 1.1.1.93) are absent in SSF of SAV (Figure S6). As a result, the in‐degree of tartrate node is only 1 (Figure 3, and Figure S8), and the only precursor of tartrate in SAV fermentation is oxaloacetate.

### 3.5 Distribution of microbes in organic acid metabolism

**FIGURE 5:**
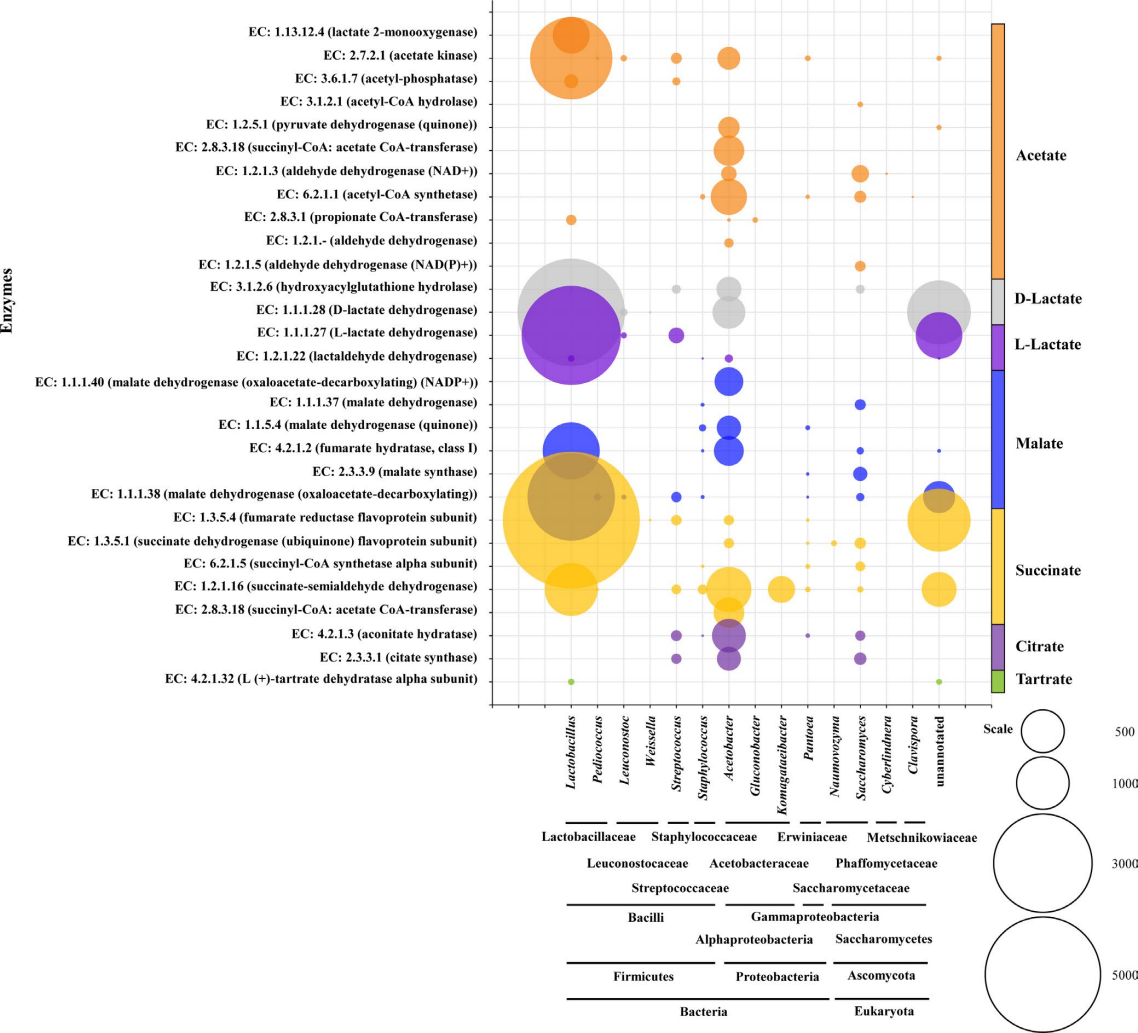
Taxonomic distribution and enzyme reads for organic acids in microbial community. The diameter of bubble correlates with the read number of enzymes

Organic acids are considered as the building block for most chemical substances produced by microbial processing (Sauer et al., 2008). During SAV fermentation, the metabolism of organic acids involves various microorganisms (Figure 5). Acetate is the principal component of vinegar and is primarily applied for flavoring (Mani‐López et al., 2012). Generally, in vinegar fermentation, acetate is mainly formed by *Acetobacter* (Panda et al., 2016). In the present work, enzymes involved in pathways of acetaldehyde to acetate (EC: 1.2.1.3, EC: 1.2.1.‐), acetyl‐CoA to acetate (EC: 2.8.3.18), and acetyladenylate to acetate (EC: 6.2.1.1) are all mainly derived from *Acetobacter* (Figure 5). However, acetyl‐P is transformed to acetate mainly by *Lactobacillus*. Similarly, the conversion of L‐lactate to acetate also only exists in *Lactobacillus*. In addition, some enzymes with low reads from *Saccharomyces*, *Streptococcus*, and *Pantoea* have also been found in the metabolism of acetate, providing more supplement for the previous research on microbial diversity related to acetate metabolism in cereal vinegar fermentation (Wu et al., 2017).

Lactate can be formed by several bacteria through anaerobic fermentation (Demichelis et al., 2017). Different from the fermentation process of Zhenjiang aromatic vinegar (Wu et al., 2017), the metabolism of lactate in SAV fermentation is not only through lactoylglutathione, but also through pyruvate and lactaldehyde (Figure 3), resulting in various enzymes and multiple microbiota involved (Figure 5). *Lactobacillus* is the dominant microorganism in the lactate metabolism, followed by *Acetobacter* (Figure 5). In the previous study, *Lactobacillus,* such as *Lactobacillus fermentum*, *Lactobacillus plantarum*, *Lactobacillus casei*, and *Lactobacillus helveticus*, and AAB, such as *Acetobacter pasteurianus* and *Acetobacter aceti*, were proved to possess the ability to produce lactate, which were isolated in SAV fermentation (Wu et al., 2012; Zheng et al., 2018).

Malate is the intermediate of TCA cycle, which is a nonvolatile organic acid with relatively high content in vinegar and is used as an acidulant, flavor enhancer, and one component of antimicrobial agents (Chi et al., 2016). During SSF of SAV, pyruvate and fumarate can be transformed to malate by the co‐effect of *Lactobacillus* and *Acetobacter* (Figure 5). However, malate dehydrogenase (EC: 1.1.1.37, EC: 1.1.5.4) involved in the oxaloacetate to malate pathway is mainly originated from *Acetobacter* and *Saccharomyces*. During saké fermentation, succinate combined with malate is produced by *Saccharomyces cerevisiae*, which confers an umami and refreshing taste for sake (Nakayama et al., 2012). In the present study, *Saccharomyces* is also involved in the metabolism of succinate. Additionally, succinate could be synthesized by Acetobacteraceae, including *Acetobacter* and *Komagataeibacter*. However, fumarate reductase (EC: 1.3.5.4) from genus of *Lactobacillus* has a number of reads as high as 6,926, resulting in the dominant role of *Lactobacillus* in the succinate metabolism.

Citrate is another TCA cycle intermediate, and numerous microorganisms have been reported to accumulate it (Soccol et al., 2006). During SSF of SAV, *Acetobacter* is the main genus for citrate metabolism, followed by *Streptococcus* and *Saccharomyces* (Figure 5). Tartrate was reported to be the main organic acid in wine, which has an important influence on taste, mouthfeel, and aging potential of wine (DeBolt et al., 2006). Moreover, it is generally believed that ascorbic acid is the main precursor of tartrate biosynthesis (Shangguan et al., 2015). However, in SSF of SAV, the synthesis of tartrate is only related to the oxaloacetate pathway and is carried out with a small amount of *Lactobacillus* (Figure 5).

## 4 CONCLUSIONS

Through metagenomics and bioinformatics technology, the metabolic network of key organic acids with TAV higher than 1 in SSF of SAV was reconstructed. Pyruvate is the core compound in OAMN. The metabolic pathway of acetate played a pivotal role in this network, and *Acetobacter* and *Lactobacillus* are the main genera that participate in organic acid metabolism in SSF of SAV.

## CONFLICT OF INTEREST

The authors declare that they do not have any conflict of interest.

## Supporting information

App S1

## Data Availability

Data are available on request from the corresponding author.
