# Supplementary material for: Unraveling the metabolic network of organic acids in solid‐state fermentation of Chinese cereal vinegar
Source: Food Sci Nutr. 2021 Jun 18;9(8):4375–84. doi: 10.1002/fsn3.2409 (PMC8358386; doi:10.1002/fsn3.2409)
Supplement: Supplementary file 1 — App S1 [file FSN3-9-4375-s001.docx]

**Table S1** Summary of metagenomic sequence assembly information

| Feature | Data |
| --- | --- |
| Type of sequencing | Illumine Hiseq 2000 |
| Smallest contig (bp) | 200 |
| Largest contig (bp) | 97470 |
| Number of contigs | 83968 |
| N50 (bp) | 935 |
| Total number of bases pre-assembly | 4.95×10^6^ |
| G+C content (%) | 41.51 |

**Table S2** Top 15 hub node of the metabolic networks ranked by the betweenness centrality

|  | Node | Node Type | Betweenness Centrality |
| --- | --- | --- | --- |
| 1 | Pyruvate | compound | 0.11085546 |
| 2 | Acetyl-CoA | compound | 0.08946366 |
| 3 | Acetate | compound | 0.08151381 |
| 4 | 2.8.3.18 (succinyl-CoA: acetate CoA-transferase) | enzyme | 0.07506083 |
| 5 | Ethanal | compound | 0.04530308 |
| 6 | 4.1.1.1 (pyruvate decarboxylase) | enzyme | 0.04345181 |
| 7 | Ethanol | compound | 0.04080715 |
| 8 | 4.1.1.2 (oxalate decarboxylase) | enzyme | 0.03333333 |
| 9 | 1.2.1.10 (acetaldehyde dehydrogenase (acetylating) | enzyme | 0.03212737 |
| 10 | 1.2.5.1 (pyruvate dehydrogenase (quinone) | enzyme | 0.02721358 |
| 11 | PEP (phosphoenolpyruvate) | compound | 0.02221517 |
| 12 | 2.3.1.54 (formate C-acetyltransferase) | enzyme | 0.02025812 |
| 13 | 1.2.7.1 (pyruvate ferredoxin oxidoreductase alpha subunit) | enzyme | 0.02025812 |
| 14 | TPP (Thiamin diphosphate) | compound | 0.02017878 |
| 15 | 2.7.1.40 (pyruvate kinase) | enzyme | 0.01798371 |

**Table S3** Node degrees of organic acids in the metabolic networks

|  | Node | Degree | In-degree |
| --- | --- | --- | --- |
| 1 | Acetate | 11 | 11 |
| 2 | Malate | 6 | 6 |
| 3 | Succinate | 5 | 5 |
| 4 | L-Lactate | 4 | 2 |
| 5 | D-Lactate | 3 | 2 |
| 6 | Citrate | 2 | 2 |
| 7 | Tartrate | 1 | 1 |


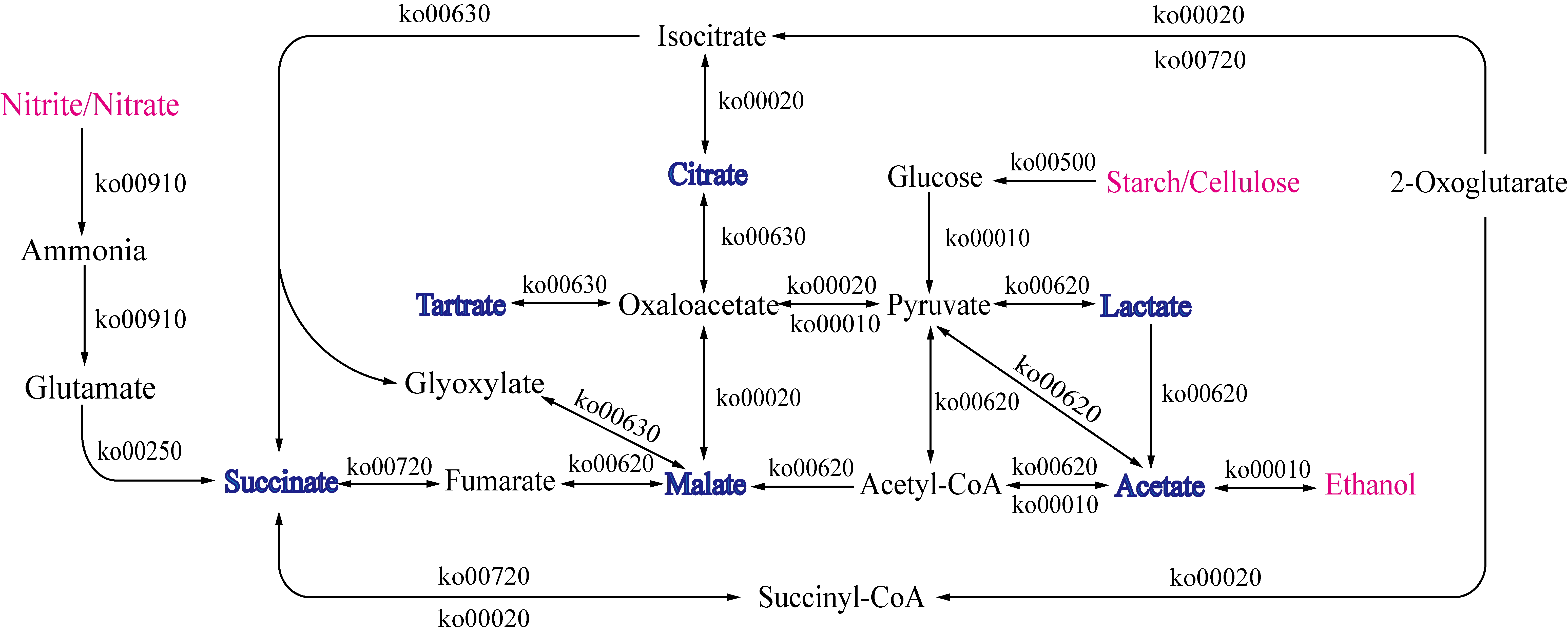


**Figure S1.** Metabolic pathways for substrate breakdown and main organic acids formation in the microbial community of SAV. (Red represents substrates, whereas blue denote main organic acids)


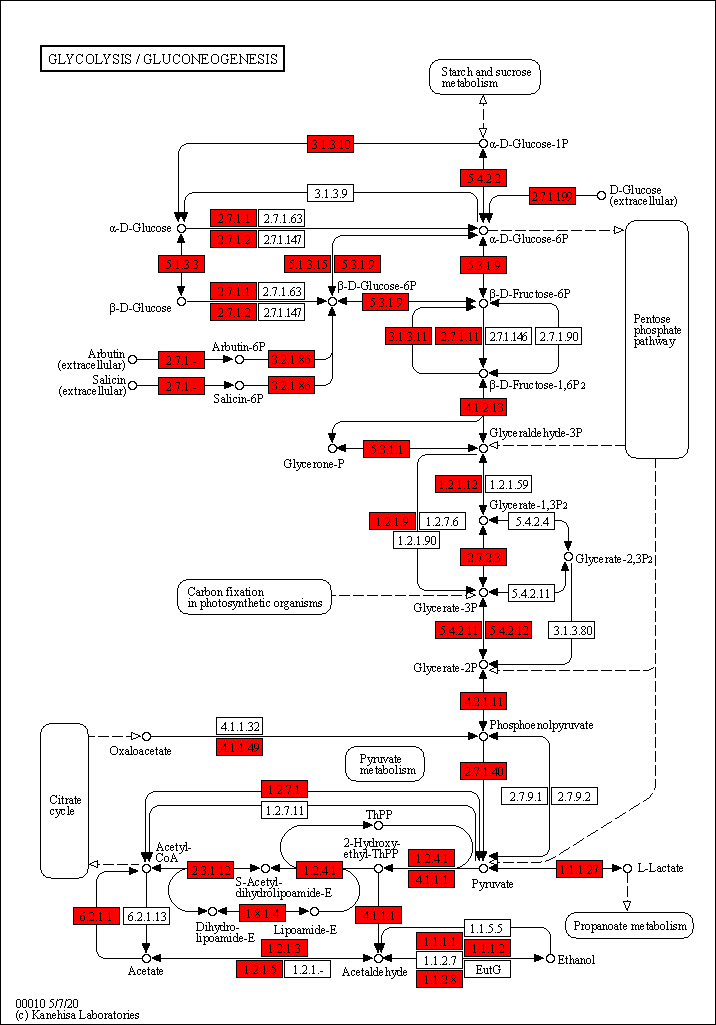


**Figure S2** Enzymes annotated in the pathway of ko00010

**
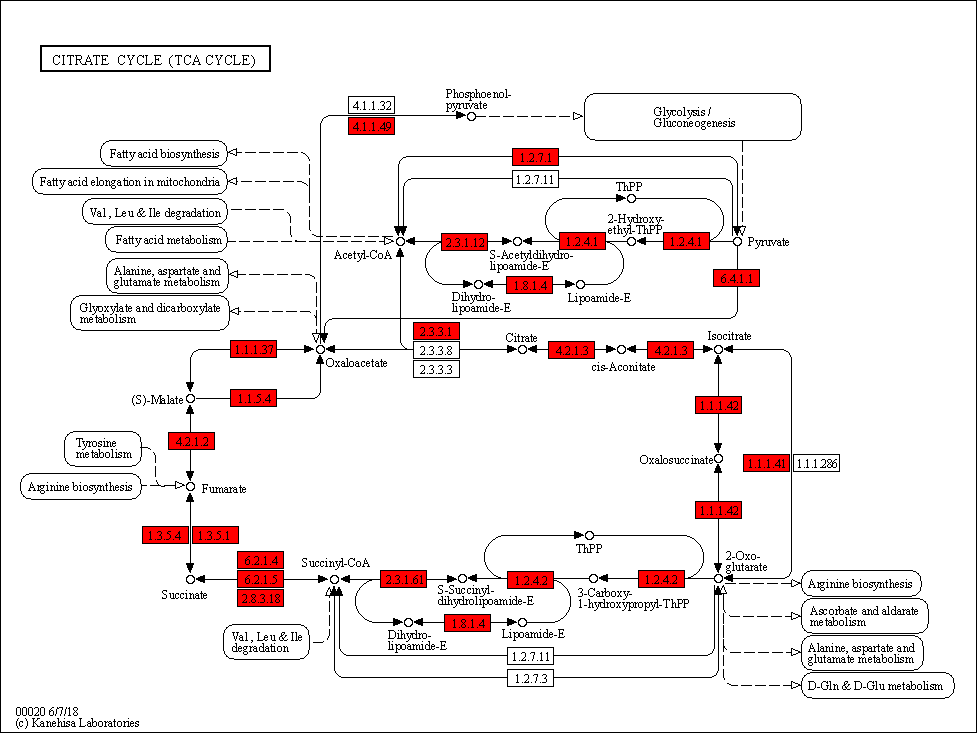
**

**Figure S3** Enzymes annotated in the pathway of ko00020

**
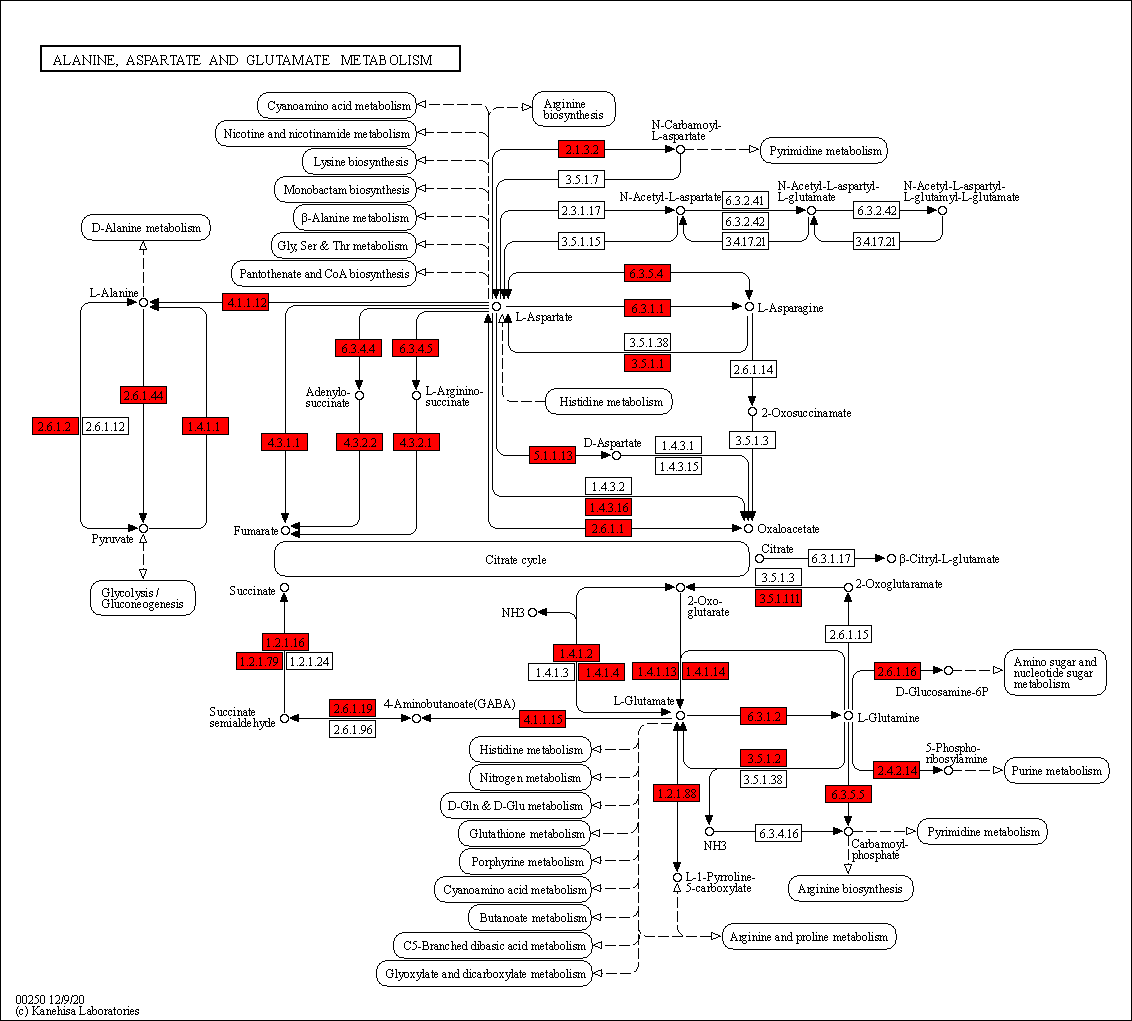
**

**Figure S4** Enzymes annotated in the pathway of ko00250

**
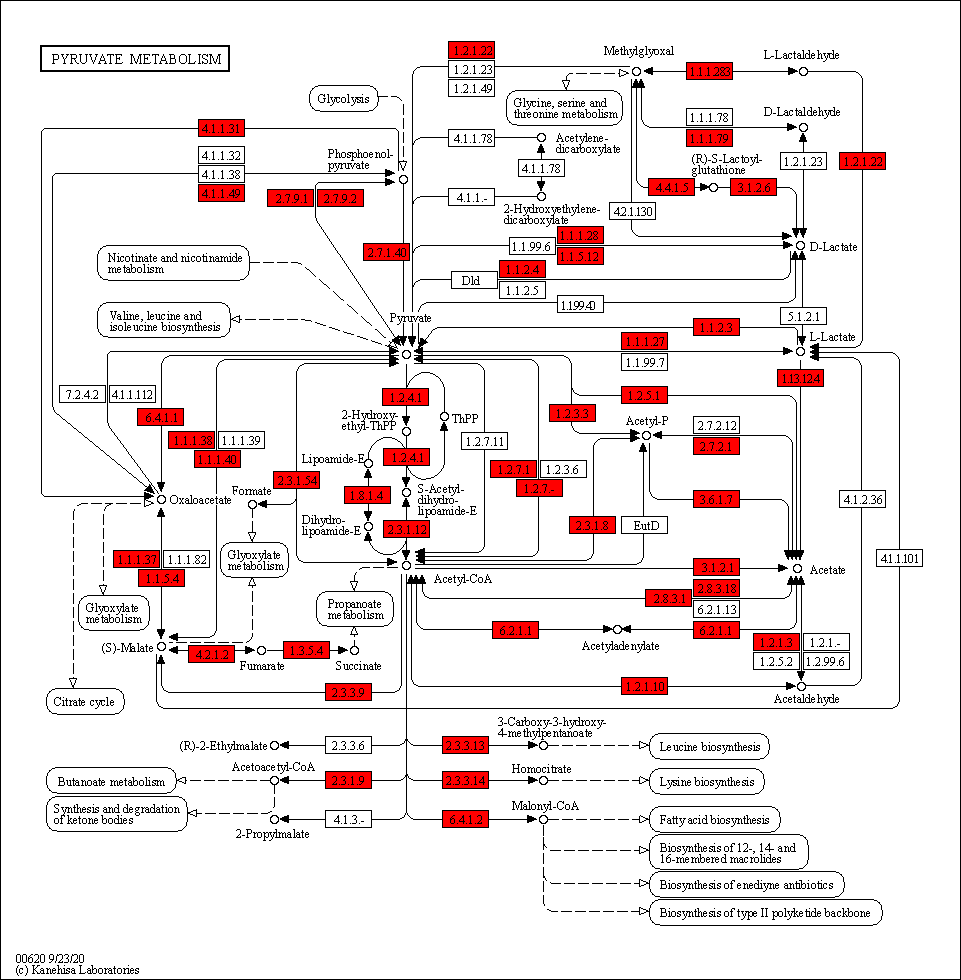
**

**Figure S5** Enzymes annotated in the pathway of ko00620

**
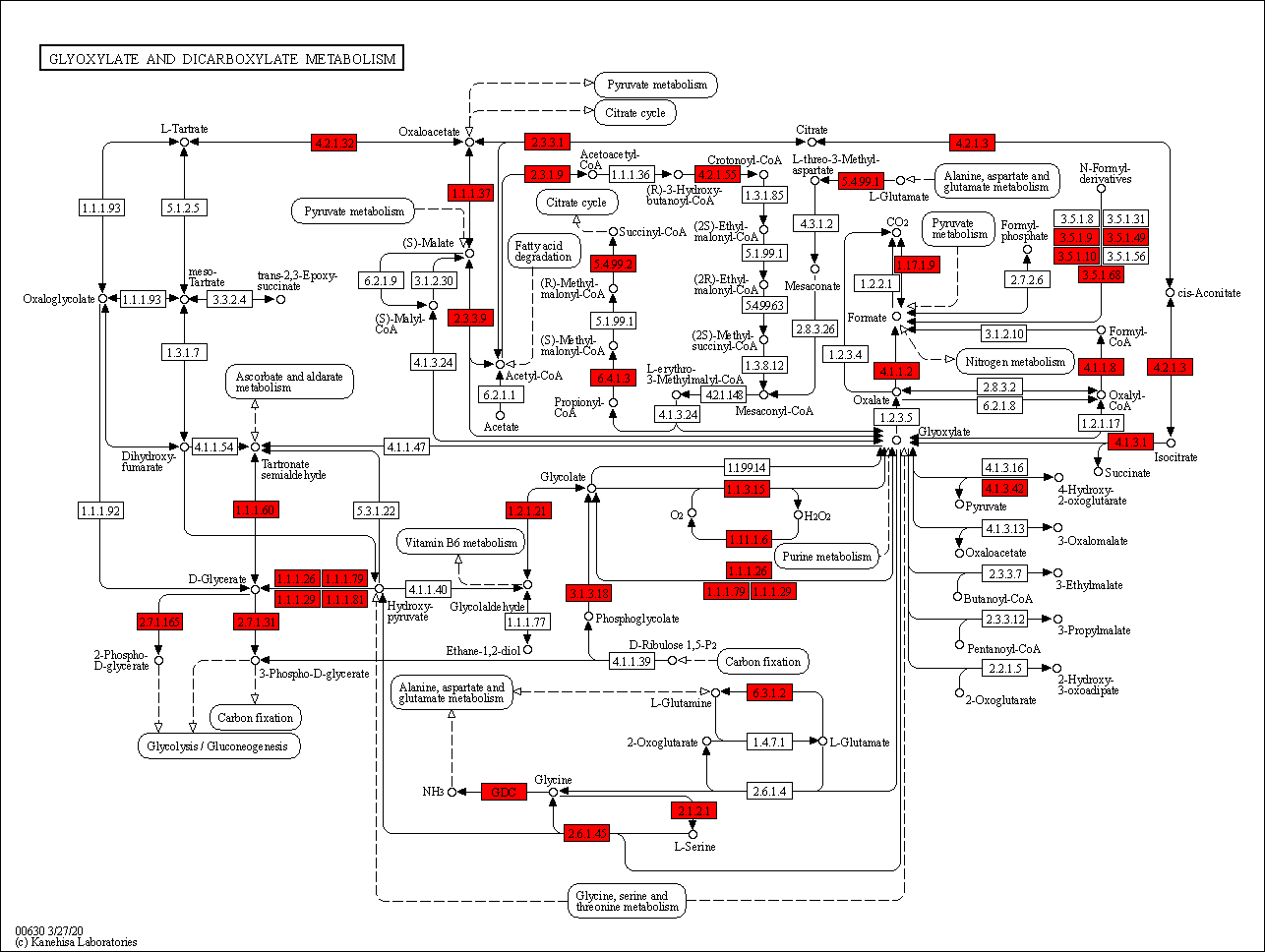
**

**Figure S6** Enzymes annotated in the pathway of ko00630

**
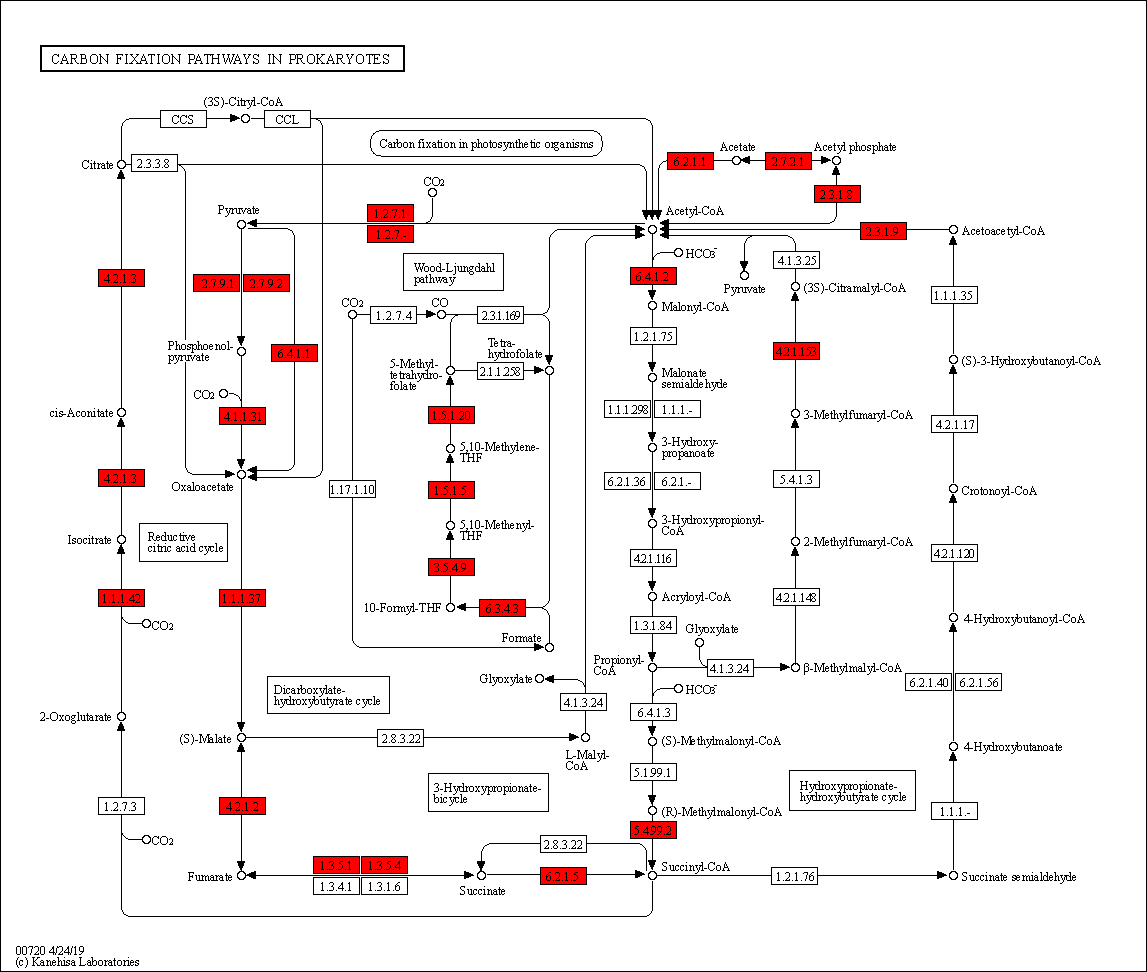
**

**Figure S7** Enzymes annotated in the pathway of ko00720





**Figure S8** Specific information of metabolic pathways of 6 organic acids
